# Strengthening peptide-based drug activity with novel glyconanoparticle

**DOI:** 10.1371/journal.pone.0204472

**Published:** 2018-09-27

**Authors:** Jordan D. Lewicky, Alexandrine L. Martel, Nya L. Fraleigh, Amanda Boraman, Thi M.-D. Nguyen, Peter W. Schiller, Tze Chieh Shiao, René Roy, Hoang-Thanh Le

**Affiliations:** 1 Health Sciences North Research Institute, Sudbury, Ontario, Canada; 2 Laboratory of Chemical Biology and Peptide Research, Clinical Research Institute of Montreal, Montreal, Quebec, Canada; 3 Department of Pharmacology and Physiology, University of Montreal, Montreal, Quebec, Canada; 4 Glycovax Pharma Inc., Montreal, Quebec, Canada; 5 Northern Ontario School of Medicine, Medicinal Sciences Division, Sudbury, Ontario, Canada; 6 Department of Chemistry and Biochemistry, Laurentian University, Sudbury, Ontario, Canada; 7 Department of Biology, Laurentian University, Sudbury, Ontario, Canada; Helsingin Yliopisto, FINLAND

## Abstract

The therapeutic application of peptide-based drugs is significantly limited by the rapid proteolytic degradation that occurs when in blood. Encapsulation of these peptide structures within a delivery system, such as liposomes, can greatly improve both stability and target delivery. As part of our work focused on novel ambiphilic mannosylated neoglycolipids as targeted drug delivery systems, we have developed a C_14_-alkyl-mannopyranoside that forms self-assembled monodisperse liposomes. Herein, these glycoliposomes are investigated as a potential method to improve the plasma stability of peptide-based drugs. Reversed phase high-performance liquid chromatography (RP-HPLC) and mass spectrometry (MS) methods were developed to assess the *in vitro* plasma stability of two structurally diverse peptides, including the kappa opioid receptor selective antagonist dynantin, and the NOD2 innate immune receptor ligand muramyl dipeptide (MDP). The RP-HPLC methods developed were able to resolve the peptides from background plasma contaminants and provided suitable response levels and linearity over an appropriate concentration range. Both compounds were found to be significantly degraded in rat plasma. Increasing degrees of both entrapment and stabilization were noted when dynantin was combined with the C_14_-alkyl-mannopyranoside in increasing peptide:glycoside ratios. The combination of MDP with the glycolipid also led to peptide entrapment, which greatly improved the plasma stability of the peptide. Overall, the results clearly indicate that the stability of peptide-based structures, which are subject to degradation in plasma, can be greatly improved via entrapment within C_14_-alkyl-mannopyranoside-bearing glycoliposomes.

## Introduction

Generally, peptides have low toxicity, high specificity and high affinity to their targets, making them interesting molecules for drug development [1–4]. However, the therapeutic potential of peptides is limited due to poor bioavailability, poor absorption through membranes, cleavage by proteolytic enzymes, and rapid elimination by both the reticuloendothelial system and by renal filtration [5–8]. This rapid metabolism and elimination results in an insufficient half-life *in vivo* for the peptides to reach their therapeutic target.

The stability of peptides can be measured in serum, plasma, or whole blood from humans or animals to assess the suitability of peptides as therapeutic drugs [7, 9–15]. Many potential therapeutic peptides have shown to be partially or fully degraded in biological matrices, including antibacterial peptides [7, 10], hormone peptides [9, 15], neuropeptides [16, 17], and anti-cancer peptides [18], which has ultimately limited their use as therapeutics. Enzyme degradation in the blood has led to the development of different strategies to improve peptide stability, including chemical modification, peptide self-assembly, the use of protease and peptidase inhibitors, or formulating the peptide in a drug delivery system. Chemical modifications that can be used to prevent enzyme recognition and reduce degradation include altering the N- and C-terminals, cyclisation, polymerization, and the incorporation of non-natural amino acids [2, 8, 9, 15, 16, 19–21]. However, modifications to the peptide structure carry the inherent risk that activity, receptor affinity, or receptor selectivity may be reduced or lost upon modification [20]. Peptide self-assembly into ordered nanostructures has also been shown to improve stability against degradation, and has the advantage of the carrier itself being therapeutic [22–25]. However, self-assembled peptides do not always maintain their structure *in vivo* while other issues such as size control, solubility and toxicity of the nanomaterials were not fully assessed [23, 26].

Encapsulating peptides in a delivery system can improve both peptide stability and delivery to the target without altering peptide activity [27]. Nanoparticles (NPs) have shown to improve delivery and reduce peptide degradation of hormone peptides [28, 29], cancer drug peptides [20], and antimicrobial peptides [30]. Recently, sugars, including monosaccharides and disaccharides, have been used to form glycosylated NPs [30, 31]. Lipid NPs functionalized with mannose demonstrated a high affinity to specific targets containing mannose receptors such as dendritic cells, macrophages, intestinal M-cells, and cancer cells [32–39]. Glycolipids have also been previously shown to increase permeability through cell membranes [40]. Specifically, mannosylated liposomes had up to a 7-fold increase in permeability across the gastrointestinal wall compared to conventional liposomes [41]. Liposomes are particularly interesting as a delivery system due to their ambiphilic nature that mimics the phospholipid membrane of cells, which allows the incorporation of both hydrophilic and lipophilic drugs.

**Fig 1:**
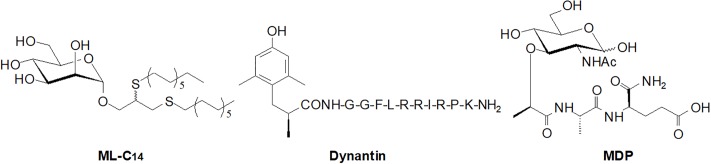
Structures of C_14_-alkyl-mannopyranoside (ML-C_14_), KOR antagonist peptide dynantin, and muramyl dipeptide (MDP).

As part of our efforts towards the development of particle-based delivery systems, we have previously investigated a library of novel ambiphilic neoglycolipids that form monodisperse liposomes through self-assembly when added to water [42]. From this library, a C_14_-alkyl-mannopyranoside (ML-C_14_, Fig 1) emerged as a promising candidate in the development of sugar-based nanoparticles. Here, we investigated the ability of the ML-C_14_ liposomes to entrap two structurally diverse peptides and protect them from degradation in plasma. These peptides were selected as models as they are both known to have plasma stability issues that would potentially hinder our efforts to use these peptides in further investigations. The first peptide is dynantin (Fig 1), which is a dynorphin A analogue with potent selectivity and antagonistic activity at the kappa opioid receptor (KOR) developed in Schiller’s research group [43]. KOR antagonists show promise as potential treatments for addiction and its associated depression that do not cause dependence, or show the high relapse rates associated with current treatment options [44–46]. The prototypical KOR antagonist nor-binaltorphimine (nor-BNI) is very stable in the body, however, its long lasting effects limit its clinical use [47–49]. Peptide structures that are capable of binding to the KOR, including dynorphin A analogues, are known to be less stable in the body and provide effects that are more transient [46]. Dynantin has the same chemical structure as dynorphin A (1–11)-NH_2_ but with a (2S)-2-methyl-3-(2,6-dimethyl-4-hydroxyphenyl)propanoic acid [(2S)-Mdp] group replacing the Tyr^1^ residue [43], and contains many of the same proteolytic cleavage sites as dynorphin A, which itself is not stable in plasma [17, 50]. In addition to poor blood stability, poor diffusion through the blood brain barrier (BBB) to access KORs is an additional limitation for the use of dynorphin A and dynorphin A analogues in a clinical setting [51]. The second model peptide was muramyl dipeptide (MDP, Fig 1), which is a 2 amino acid glycopeptide component of bacterial cell wall peptidoglycan. MDP has been shown to activate NOD2, the innate immune receptor, and has been investigated for its adjuvant activity [52]. Limitations towards the clinical use of MDP include poor penetration of macrophages [53], pyrogenicity [54], and rapid urinary excretion when administered as an aqueous solution [55]. MDP has also been shown to be degraded in rat plasma into its three individual structural components, N-acetylmuramic acid, L-alanine, and D-isoglutamine [53]. In this preliminary study, the stabilities of these two peptides, alone and entrapped in ML-C_14_ glycoliposomes, were assessed *in vitro* over time in rat plasma by RP-HPLC. Entrapping dynantin or MDP in our glycoliposomal delivery system aims to improve the plasma stability of these peptides, with the ultimate goal being the improvement of *in vivo* peptide bioavailability.

## Material & methods

### Reagents

The preparation of dynantin followed the previously published methods, and the compound was stored at -20 °C as a lyophilized powder [43]. Purity was determined by HPLC to be ≥ 98%. A commercial source of MDP was obtained as a lyophilized powder (≥98%, Sigma Aldrich, St. Louis MO). Stock solutions of each peptide were generated in ddH_2_O (5 μg/μL) and stored as 12 μL aliquots at -80 °C.

The preparation of ML-C_14_ followed the previously published methods [42], and the compound was kept stored at -20 °C as a lyophilized powder. Purity was determined to be ≥ 95% as indicated by thin-layer chromatography and nuclear magnetic resonance spectroscopic analyses. A stock solution (50 μg/μL) was prepared for *in vitro* analyses by dissolving the compound in tetrahydrofuran (THF, HPLC grade, Fisher Scientific, Fairlawn NJ), and stored at -20 °C. When required, less concentrated solutions were prepared by dilution of this stock in THF.

### Rat Plasma collection

Female Sprague Dawley rats were purchased from Charles River (QC, Canada). Rats were housed at the Laurentian University Animal Care Facility and supplied with food and water *ad libitum*. All experimental protocols were approved by the Health Sciences North Research Institute biosafety committee and the Laurentian University animal care committee. Blood was collected from rats under a surgical plane of anesthesia using isoflurane via cardiac puncture. Rats were then euthanized by excess isoflurane in addition to the severing of the descending aorta and cutting of the diaphragm. Blood was immediately mixed with an ethylenediaminetetraacetic acid solution (Fisher Scientific, Fairlawn NJ,0.1 M, 5 mM final concentration) and stored on ice until being centrifuged at 2,100 x G and 4 °C for 10 minutes. The top plasma layer was removed, aliquoted and stored at -80 °C.

### HPLC conditions

All analyses were performed using a Shimadzu Prominence series HPLC system (Shimadzu Corporation, Kyoto, Japan), equipped with a LC-20AB binary pump (Serial: L20124200883), SIL-20A HT autosampler (Serial: L20345256104), CTO-20AC temperature controlled column oven (Serial: L2021525077), and CBM-20A communications bus (Serial: L20235154327). All equipment was controlled by Shimadzu LabSolutions Lite software version 5.71 SP2. For separation, an Ultra C18 column, 3 μm, 50 x 4.6 mm (RESTEK Corporation, Bellefonte, PA) was used.

Dynantin samples were analyzed at a constant solvent flow rate of 0.7 mL/min at 35 °C using a binary gradient (Table 1). Solvent A consisted of a 25% solution of acetonitrile (HPLC grade Fisher Scientific) in ddH_2_O (0.2 μm filtered) and solvent B consisted of acetonitrile, with each solvent containing 0.1% trifluoroacetic acid (v/v, protein sequencing grade, Sigma Aldrich, Fairlawn NJ).

**Table 1:** Solvent gradient program for the analysis of dynantin plasma stability using 25% acetonitrile in water (A) and acetonitrile (B), both with 0.1% trifluoroacetic acid (v/v).

Time (min)	Solvent	
	A (%)	B (%)
0	100	0
15	40	60
18	20	80
26	20	80
30	100	0
40	100	0

MDP samples were analyzed at a constant solvent flow rate of 1.0 mL/min at 35 °C using a binary gradient (Table 2). Solvent A consisted of ddH_2_O (0.2 μm filtered) and solvent B consisted of methanol (MeOH) (HPLC grade, Fisher Scientific, Fairlawn NJ), with each solvent containing 0.1% formic acid (v/v, LC/MS grade, Fisher Scientific).

**Table 2:** Solvent gradient program for the analysis of MDP plasma stability using water (A) and methanol (B), both with 0.1% formic acid (v/v).

Time (min)	Solvent	
	A (%)	B (%)
0	98	2
5	98	2
10	40	60
15	40	60
20	98	2
27	98	2

### Dynantin standard curve

The dynantin stock solution (5 μg/μL) was diluted to varying degrees in ddH_2_O and then analyzed by RP-HPLC (8 μL injections, in duplicate) to generate a 5-point standard curve covering a 50–800 ng injection mass range.

### Peptide plasma stability analysis

Peptide stability was investigated by combining dynantin and MDP aliquots with thawed rat plasma (88 μL), followed by incubation at 37 °C in a heating mantle (VWR Scientific) for varying lengths of time before being stored at -80 °C. The degree of peptide degradation was analyzed by RP-HPLC, in which samples were thawed, thoroughly mixed and diluted in ddH_2_O (1/10 for Dynantin, 1/5 for MDP) before analysis (8 μL injections, in duplicate). Stability is represented as the percentage of peptide remaining relative to the amount determined at time point zero (T-zero).

### Peptide entrapment

Glycoliposomal entrapment was investigated by combining peptide aliquots with varying amounts of ML-C_14_ (in THF, 20 μL total addition) in ddH_2_O to a final volume of 100 μL. The mixtures were gently vortexed for 5 minutes, after which solids were pelleted by centrifugation at 14,000 rpm and 20 °C for 10 min. Supernatants were carefully removed for analysis of the levels of non-entrapped peptide that remained (8 μL injections, in duplicate). The degree of peptide entrapment is represented as the percentage of entrapped peptide relative the amount determined in respective control samples comprised of peptide and THF devoid of glycolipid.

### Peptide with mannose lipid plasma stability analysis

Peptide stability in combination with ML-C_14_ was investigated using a modified version of the above noted procedure. Aliquots of dynantin or MDP were first combined with varying amounts of ML-C_14_ (in THF, 20 μL total addition), and then thoroughly mixed before the addition of thawed rat plasma (88 μL). A control sample was also prepared by combining each peptide with THF alone (20 μL) and plasma (88 μL). Samples were incubated at 37 °C in a heating mantle for varying lengths of time before being stored at -80 °C. For HPLC analysis, samples were thawed, thoroughly mixed, diluted in MeOH to destroy liposome particles (1/10 for dynantin, 1/5 for MDP), and solids pelleted by centrifugation at 10,000 rpm and 20 °C for 5 min. The supernatants were carefully removed for analysis (8 μL injections, in duplicate). Stability is represented as the percentage of peptide remaining relative to the amount determined at T-zero.

### Mass spectrometry analysis

All analyses were carried out in the lab of Dr. R.J. Neil Emery in the Department of Biology at Trent University (Peterborough, Canada).

For the analysis of dynantin plasma degradation, a dynantin solution (120 μL, 10 μg/μL in ddH_2_O) was combined with thawed rat plasma (600 μL) and the resulting solution incubated at 37 °C for 24 hours. The sample was then diluted 1/10 with anhydrous ethanol (Fisher Scientific, Fairlawn NJ) and the mixture gently vortexed for 5 minutes, after which solids were pelleted by centrifugation at 14,000 rpm and 20 °C for 10 min. The supernatant was carefully removed, concentrated under vacuum, and the remaining solution (≈500 μL) divided into two portions. One portion was frozen at -80 °C overnight before being lyophilized (Alpha 1–2 LD, CHRIST, Germany), and the other portion was fractionated into individual degradation product peaks via the HPLC conditions outlined above (duplicate 100 μL injections). Fractions for each peak were pooled, frozen at -80 °C overnight, and lyophilized. The lyophilized samples were analyzed after reconstitution by direct injection using an Orbitrap Q Exactive (Thermo Fisher Scientific, Bremen, Germany) [56]. Both negative [M-H]^-^ and positive [M+H]^+^ modes were examined using a HESI-II source immediately prior to MS analysis. While both polarities were examined, the negative ion mode provided the best ionization. A consistent flow rate of 50 μL/min was used, with the electrospray needle voltage maintained at 4 kV, ultrapure nitrogen sheath gas flow kept at 12 L/min, and the heated metal capillary temperature set to 320 °C. Resolving power was set to 140,000 (FWHM at *m/z* 200) with an overall accuracy of <2 ppm and automatic gain control target of 5 x 10^6^ between the range of *m/z* of 200 and 2000.

For the analysis of MDP, individual peaks were fractionated via the HPLC conditions outlined above (quintuplicate 50 μL injections of stock solution). Fractions for each peak were pooled, frozen at -80 °C overnight, and lyophilized. A lyophilized aliquot (≈100 μg) of the stock solution was also prepared. Reconstituted MDP samples were analyzed by electrospray ionization liquid chromatography-tandem mass spectrometry using a Dionex Ultimate 3000 HPLC (Oakville, Canada) with a Kinetex C18 column 2.6 μm, 50 x 2.1 mm (Phenomenex, Torrance CA) connected to an AB SCIEX 5500 API mass spectrometer (Concord, Canada) [57]. Samples were analyzed at a constant solvent flow rate of 0.2 mL/min at 35 °C using an isocratic mixture of 10% MeOH in water with 0.1% formic acid.

### Statistical analyses

Data are represented as ± SEM of three separate experiments. Statistical analyses in the form of one way Anova with Tukey post hoc tests were performed using Graph Pad Prism 5.

## Results

An optimized reverse phase gradient HPLC method was developed for the analysis of dynantin plasma stability based on the previously reported methods [43]. The method provided excellent results in terms of retention time, peak area reproducibility, detection sensitivity, and resolution between the peptide and the plasma background peaks (S1 Fig). Using this method, a standard curve was generated for dynantin (S2 Fig) which showed consistent linearity over a concentration range appropriate to the intended stability studies (50–800 ng injection mass).

**Fig 2:**
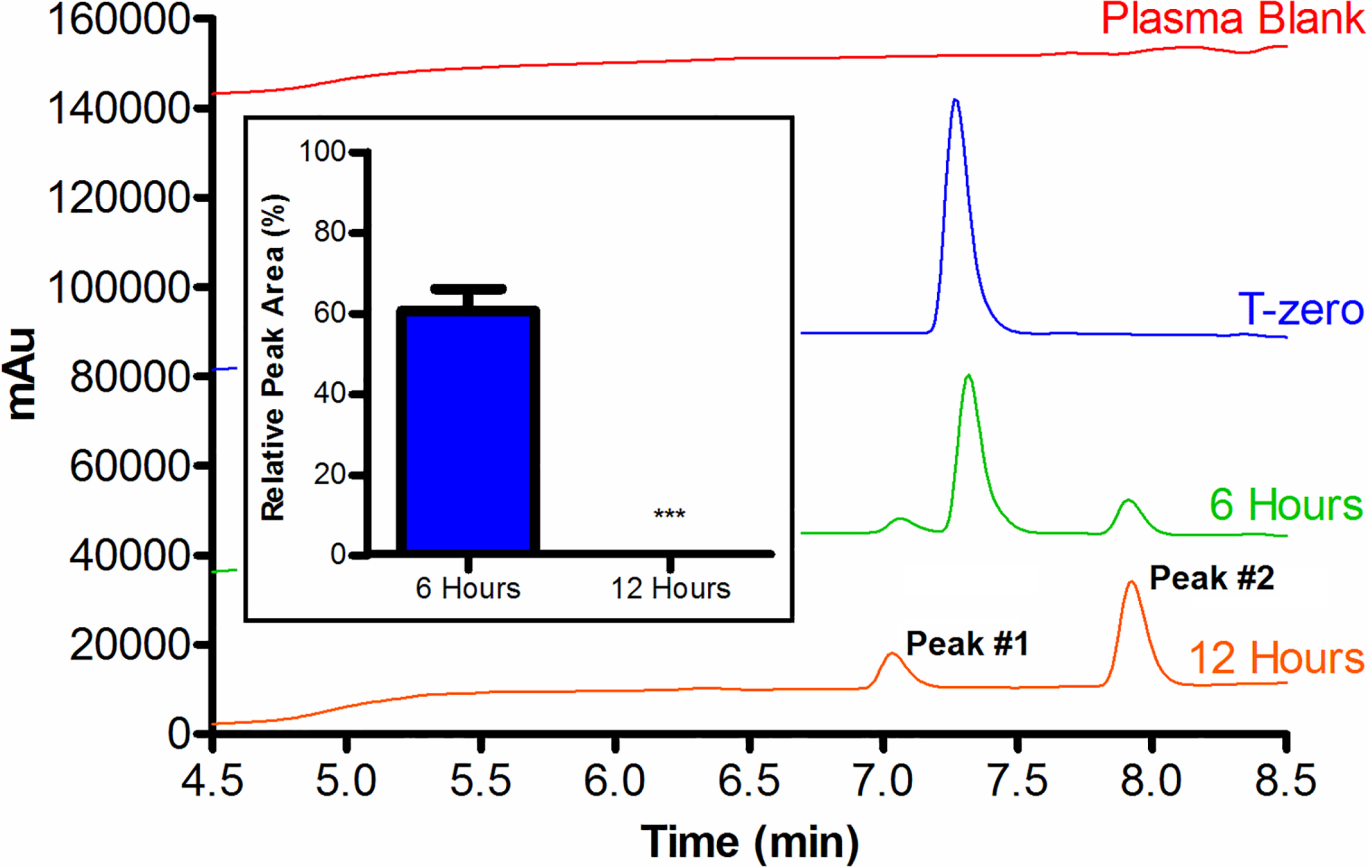
Degradation of dynantin in plasma. Dynantin was incubated in rat plasma at 37 °C. Levels of dynantin remaining after various time points were analyzed by RP-HPLC in the presence of 0.1% trifluoroacetic acid and detected by absorbance at 210 nm. The baselines of certain chromatograms have been shifted to a higher absorbance for the purpose of clarity. Peptide amounts were calculated relative to the quantities determined at time point zero, and data shown are the average ± SEM of three separate experiments (inset). *** p < 0.001 as compared to 6 hours.

The stability of the dynantin peptide in rat plasma was assessed over a 12 hour window (Fig 2). A considerable degradation of the dynantin (39 ± 4%) and the appearance of two new closely eluting degradation product peaks can be seen after 6 hours of incubation. A further 6 hours of incubation leads to complete dynantin degradation and increasing levels of both degradation products. The plasma degradation products of Dynantin were subjected to preliminary direct injection MS analysis as both the crude mixture, and individual peaks after HPLC fractionation (S3 Fig). Three degradation products have been identified in the crude mixture ([M-H]^-^ = 581.300 from [(2S)-Mdp-G-G-F-L-OH], [M-H]^-^ = 737.404 from [(2S)-Mdp-G-G-F-L-R-OH], [M-H]^-^ = 893.509 from [(2S)-Mdp-G-G-F-L-R-R-OH]) which result from proteolytic cleavage centered around and in between the two sequential arginine residues. The early eluting degradation product peak (T_R_: 7.02 min) appears to be a mixture of all three fragments, while the late eluting peak (T_R_: 7.92 min) appears to be comprised solely of the one fragment resulting from cleavage between the arginines. The presence of this later eluting fragment in the mass spectrum of the early eluting fraction is likely a consequence of imperfect fractionation. Peaks corresponding to the reciprocal fragments of the three degradation products identified were not observed. The most predominant peak in the mass spectrum of the crude mixture ([M-H]^-^ = 641.095) has not been identified, and this peak is also present in the spectrums of the fractionated peaks, albeit with lesser abundance.

**Fig 3:**
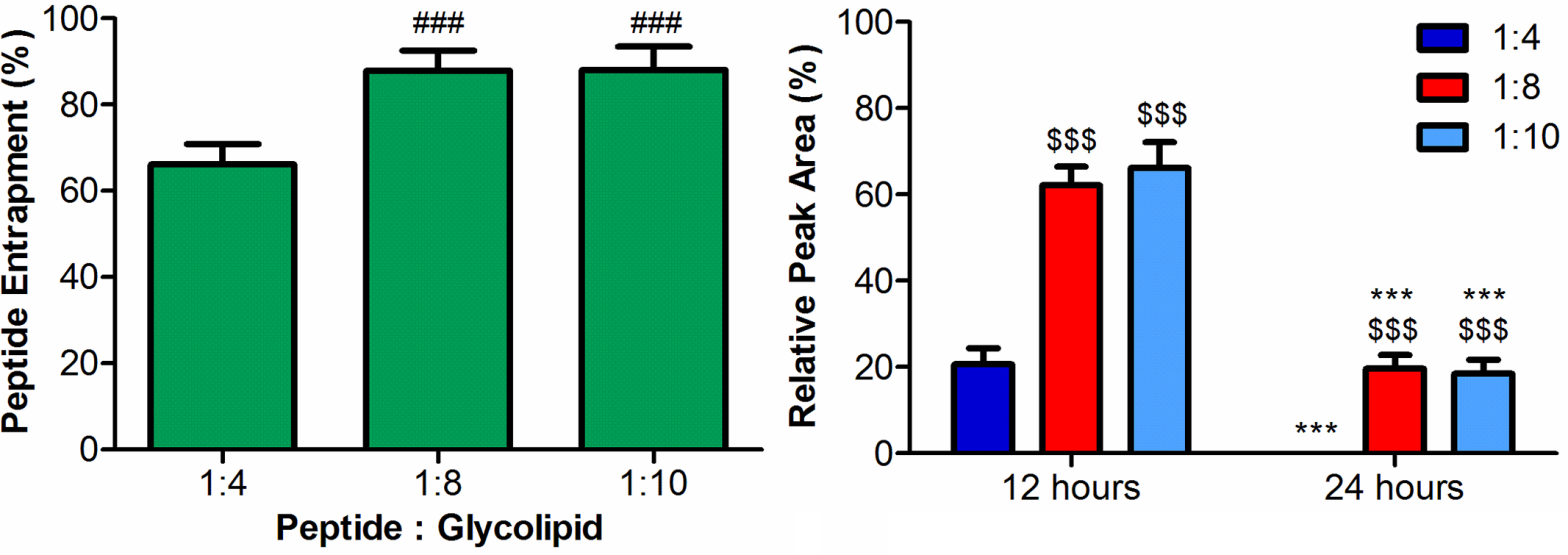
Glycoliposomal entrapment of dynantin and stability in plasma. Dynantin was combined with ML-C_14_ at different peptide: glycolipid ratios and the degree of peptide entrapment was analyzed by RP-HPLC in the presence of 0.1% trifluoroacetic acid and detected by absorbance at 210 nm. The degree of peptide entrapment is represented as the percentage of entrapped peptide relative the amount determined in control samples devoid of glycolipid. Data are shown as the average ± SEM of three separate experiments (left). ### p < 0.001 as compared to the 1:4 ratio. Combinations of dynantin and ML-C_14_ in the same ratios were incubated in rat plasma at 37 °C. Levels of Dynantin remaining after various time points were analyzed by RP-HPLC. Peptide amounts were calculated relative to the quantities determined at time point zero, and data are shown as the average ± SEM of three separate experiments (right). $ $ $ p < 0.001 as to compared to the 1:4 ratio at respective time point, *** p < 0.001 as compared to respective 12 hour time points.

The degree to which the ML-C_14_ glycoliposomes entrapped the dynantin peptide and the subsequent protection from degradation in plasma that this entrapment provided was assessed for different peptide:glycolipid ratios (Fig 3). There is a clear relationship between entrapment and the amount of glycolipid present. With the lowest peptide: glycolipid ratio tested (1:4), 66 ± 4% of the dynantin is entrapped. When the ratio of peptide:glycolipid is increased to a 1:8, dynantin entrapment improves to 88 ± 4%; with a further increase in the ratio to 1:10 not providing any significant improvements in the level of entrapment (88 ± 5%). The relationship between dynantin glycoliposomal entrapment and plasma stability was investigated over 48 hours. There was a defined correlation between the level of peptide entrapment and the degree of degradation; the 1:4 ratio of peptide:glycolipid that provided the lowest entrapment also had the lowest amount of dynantin remaining after 12 hours in plasma (21 ± 4%), and no detectable peptide after 24 hours. The 1:8 and 1:10 ratios of peptide:glycolipid, which had higher entrapment levels compared to the 1:4 ratio, also had higher levels of the peptide that remained after 12 hours (62 ± 4% for 1:8, 66 ± 6% for 1:10), in addition to having significant levels of peptide remaining after 24 hours (20 ± 3% for 1:8, 19 ± 4% for 1:10). Dynantin was found to be completely degraded in these samples after 48 hours. Similar to peptide entrapment, there were no significant differences in plasma stability between the 1:8 and 1:10 ratios of peptide:glycolipid.

**Fig 4:**
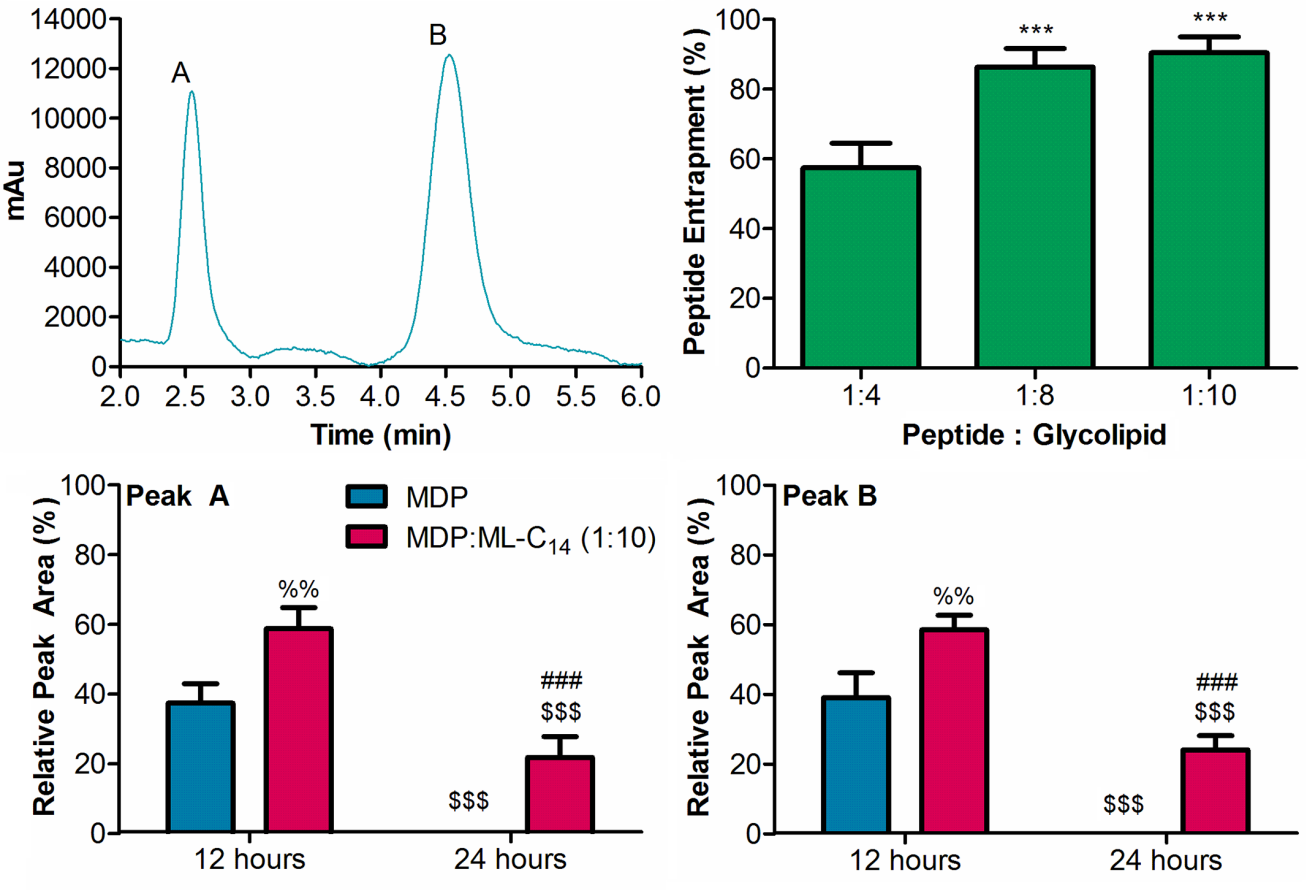
MDP RP-HPLC profile, glycoliposomal entrapment and stabilities in rat plasma. MDP was analyzed by RP-HPLC in the presence of 0.1% formic acid and detected by absorbance at 210 nm (top left). MDP was combined with ML-C_14_ at different peptide:glycolipid ratios and the degree of peptide entrapments were analyzed by RP-HPLC. The degree of peptide entrapment is based on both MDP peaks, and is represented as the percentage of entrapped peptide relative the amount determined in control samples devoid of glycolipid. Data shown are the average ± SEM of three separate experiments (top right). *** p < 0.01 compared to 1:4 ratio. MDP, alone and in combination with ML-C_14_ at a peptide:glycolipid ratio of 1:10 was incubated in rat plasma at 37 °C. Levels of each MDP peak remaining after various time points were analyzed by RP-HPLC. Peptide amounts were calculated relative to the quantities determined at time point zero, and data are shown as the average ± SEM of three separate experiments (bottom). %% p < 0.01 compared to MDP at same time point, $ $ $ p < 0.001 compared to respective 12 hour time point, ### p < 0.001 compared to MDP at same time point.

To analyze the plasma stability of MDP, a separate gradient RP-HPLC method was developed and optimized for the parameters outlined above. Interestingly, two separate and well resolved peaks with an approximate 1:2 relative abundance (based on peak area) were observed (Fig 4). MDP was further analyzed by LC/MS both alone, and after having been fractionated into the individual peaks via HPLC (S4 Fig). The two peaks present in the chromatogram of MDP produce ions of identical mass that correspond to the mass of MDP. The fractionated peaks also produce similar LC/MS profiles. When combined with ML-C_14_, both peaks were entrapped within the glycoliposomes and similar entrapment levels to those of Dynantin were noted. The lowest entrapment (58 ± 6%) was observed with a 1:4 ratio of peptide:glycolipid, and increasing this ratio to 1:8 greatly improved entrapment (86 ± 5%). No significant increase in entrapment was achieved when the peptide:glycolipid ratio was further increased to 1:10 (90 ± 4%). When incubated in rat plasma both MDP peaks were proportionally degraded. After 12 hours of incubation, there was significant degradation of the MDP (≈60%, based on both peaks), and after 24 hours it was completely degraded. When combined with ML-C_14_ at a peptide:glycolipid ratio of 1:10, plasma stability improved considerably at both the 12 hour(≈60% remaining, based on both peaks) and 24 hour (≈23% remaining, based on both peaks) time points. Complete degradation of the MDP was noted after 48 hours.

## Discussion

The highly promising therapeutic potential of peptide-based drugs is a product of their broad chemical and biological diversity, high target affinity and specificity, and low degrees of toxicity and tissue accumulation [13]. One of the consequences of the ubiquitous expression of proteolytic enzymes is that the degradation of peptide based drugs starts immediately once in an organism, that which is independent of how the peptide is administered [58]. The administration of peptide based drugs typically occurs by injection or infusion to enable systemic circulation, and in either case, blood will be the first medium the peptide encounters. Proteolytic degradation in blood is typically studied *ex vivo* by incubating the peptide in serum or plasma which contains all of the same proteases that would be encountered *in vivo*. This assay has been used as both a predictor of proteolytic lability, and as a tool to assess the efficacy of various strategies aimed at improving stability [7, 9–15]. The results of our study show that the rapid degradation of dynantin and MDP can be improved with ML-C_14_ glycoliposomal entrapment, confirming our *ex vivo* plasma stability as a useful and inexpensive tool for the primary assessment of delivery system efficacy in the progression towards *in vivo* experiments.

Tracking the degradation of dynantin over time identified that the peptide was being broken down into at least two degradation products in rat plasma (Fig 2). To the best of our knowledge this is the first example of any studies assessing the stability of dynantin in a physiological matrix. Dynorphin A (1–11) is the parent compound from which dynantin was derived, and its metabolic degradation has been well studied in a variety of plasma sources [17, 50, 59]. Across the vast majority of species, dynorphin A (1–13) is rapidly degraded, with the terminal amino acids first hydrolyzed by exo- and carboxypeptidases to form an 11-amino acid fragment which is further hydrolyzed by proteases at the Phe^4^-Leu^5^, Leu^5^-Arg^6^, Arg^6^-Arg^7^, and Ile^8^-Arg^9^ bonds. The results of our preliminary MS studies indicate that a similar degradation occurs with dynantin, and that the two sequential arginine residues in the structure are the primary points of proteolytic cleavage in rat plasma, producing three different peptide products (S3 Fig). We did not observe peaks corresponding to the reciprocal fragments of these degradation products, suggesting that these peptide fragments are subject to further proteolytic degradation within the plasma. Interestingly, the most predominant fragment observed for dynantin after proteolysis in plasma corresponds to (2S)-Mdp-G-G-F-L-R-OH ([M-H]^-^ = 737.404), indicating that the presence of the (2S)-Mdp moiety offers protection from the known proteolytic lability.

When investigating the degradation of MDP in plasma, the first interesting observation was that the compound eluted as two well resolved peaks that were equally degraded in plasma (Fig 3). The results of our LC/MS studies with MDP suggest that these peaks represent two isomers of the same molecule (S4 Fig). In fact, a previous report has outlined the RP-HPLC separation of MDP, albeit via different chromatographic conditions, in which the α- and β-D anomers of the N-acetylmuramic acid moiety within the molecule were resolved [60]. It is very likely that the two peaks we observed in our analyses represent the same two stereoisomers. This is further supported by our LC/MS results with the individual fractionated peaks from MDP. The anomerization of the sugar that is possible when in aqueous media would rapidly reform the original equilibrium that existed between the two anomers, and thereby give rise to near identical LC/MS profiles as that of the original MDP. Previous studies examining the degradation of MDP in mammalian serum indicate that the glycopeptide is first hydrolyzed into the sugar and the dipeptide components, with the dipeptide being further hydrolyzed into its individual amino acids [61]. It is very likely that the same metabolism of MDP occurred in our plasma stability analyses, and the fact that metabolite peaks were not observed is likely a consequence of the HPLC method not being suited for the analysis of such polar species.

Delivery systems which encapsulate peptides improve both stability, and delivery to the target, without impacting peptide activity in the majority of cases [27]. The ambiphilic nature of liposomes and their ability to incorporate a wide variety of different structures make them particularly interesting as a delivery system. Overall, the glycoliposomes formed through the self-assembly of ML-C_14_ are capable of entrapping both dynantin and MDP, two peptides for which the therapeutic potential is hampered by the stability issues noted herein. Importantly, this glycoliposomal entrapment of the peptides provided significant protection against degradation in plasma. For both dynantin and MDP, the degree of entrapment was found to be dependent on the ratio of peptide to glycolipid. It is natural that as the amount of glycolipid was increased, so did the amount of liposomes forming in which these peptides could be entrapped. It appeared that a maximum entrapment for either peptide was potentially reached, as increasing the peptide:glycolipid from 1:8 to 1:10 provided no significant improvements in entrapment levels. In the case of Dynantin, the degree to which stability in plasma was improved was parallel to the degree of peptide entrapment. In the development of KOR antagonists there is a defined link between stability, activity, and overall efficacy that must be obtained [46]. An antagonist that is too stable will produce long-lasting effects that are likely to have negative consequences, while an antagonist that lacks an appropriate level of stability will be degraded too quickly to be effective. An ideal KOR antagonist would be stable and active between 12 to 24 hours to be effective, and therefore we examined the stability of Dynantin up to 48 hours. Significant levels of Dynantin remained after 12 hours in plasma when entrapped within the glycoliposomes, and the protection the particles offered could be maintained up to 24 hours. These results match nicely with the desired activity window for KOR-antagonists, thereby making this a promising avenue to improve the stability, and therefore effectiveness of dynantin, while maintaining the required transient nature of KOR antagonism.

The overall results of our study indicate the ML-C_14_ glycoliposomes are effective in their ability to protect the dynantin and MDP peptides against proteolytic degradation in plasma. The use of this delivery system with other peptide-based therapeutics is possible, but will require optimization to extend the protection offered beyond 24 hours for those peptides with longer desired activity windows. What our results do not highlight for dynantin and MDP is the effect, if any, that glycoliposomal entrapment has on their bioactivity. Moreover, the metabolism of these peptides may certainly be more significant in other tissues, such as those from the liver. Future *in vivo* studies will examine entrapped peptide bioactivity as well as provide more insight as to the overall metabolism of these peptides, for which we feel the results of this study may translate into both significant reductions in peptide metabolic clearance, and subsequent improvements in efficacy.

## Supporting information

S1 Fig Dynantin and rat plasma full chromatograms. Dynantin (blue, 25 ng/μL in ddH_2_O) or rat plasma (green) were analyzed by RP-HPLC in the presence of 0.1% trifluoroacetic acid and detected by absorbance at 210 nm (8 μL injections).
(TIF)

S2 Fig Dynantin standard curve. The Dynantin stock solution was diluted to various degrees in ddH_2_O and analyzed by RP-HPLC in the presence of 0.1% trifluoroacetic acid and detected by absorbance at 210 nm. Standard curve data shown is the average ± SEM of two separate experiments.
(TIF)

S3 Fig Dynantin plasma degradation MS analysis. The plasma degradation products of dynantin were analyzed by direct injection electrospray ionization mass spectrometry in the negative ion mode as both the crude mixture (top) and individual fractionated peaks (middle and bottom), as outlined in Fig 2.
(TIF)

S4 Fig MDP LC/MS analysis. MDP was analyzed by positive mode electrospray ionization RP-HPLC-tandem mass spectrometry in the presence of 0.1% formic acid (top right). Individual MDP peaks, as outline in Fig 4 were also fractionated and subjected to the same analysis (bottom).
(TIF)
