# Supplementary figures and images for: Strengthening peptide-based drug activity with novel glyconanoparticle
Source: PLoS One. 2018 Sep 27;13(9):e0204472. doi: 10.1371/journal.pone.0204472 (PMC6160049; doi:10.1371/journal.pone.0204472)

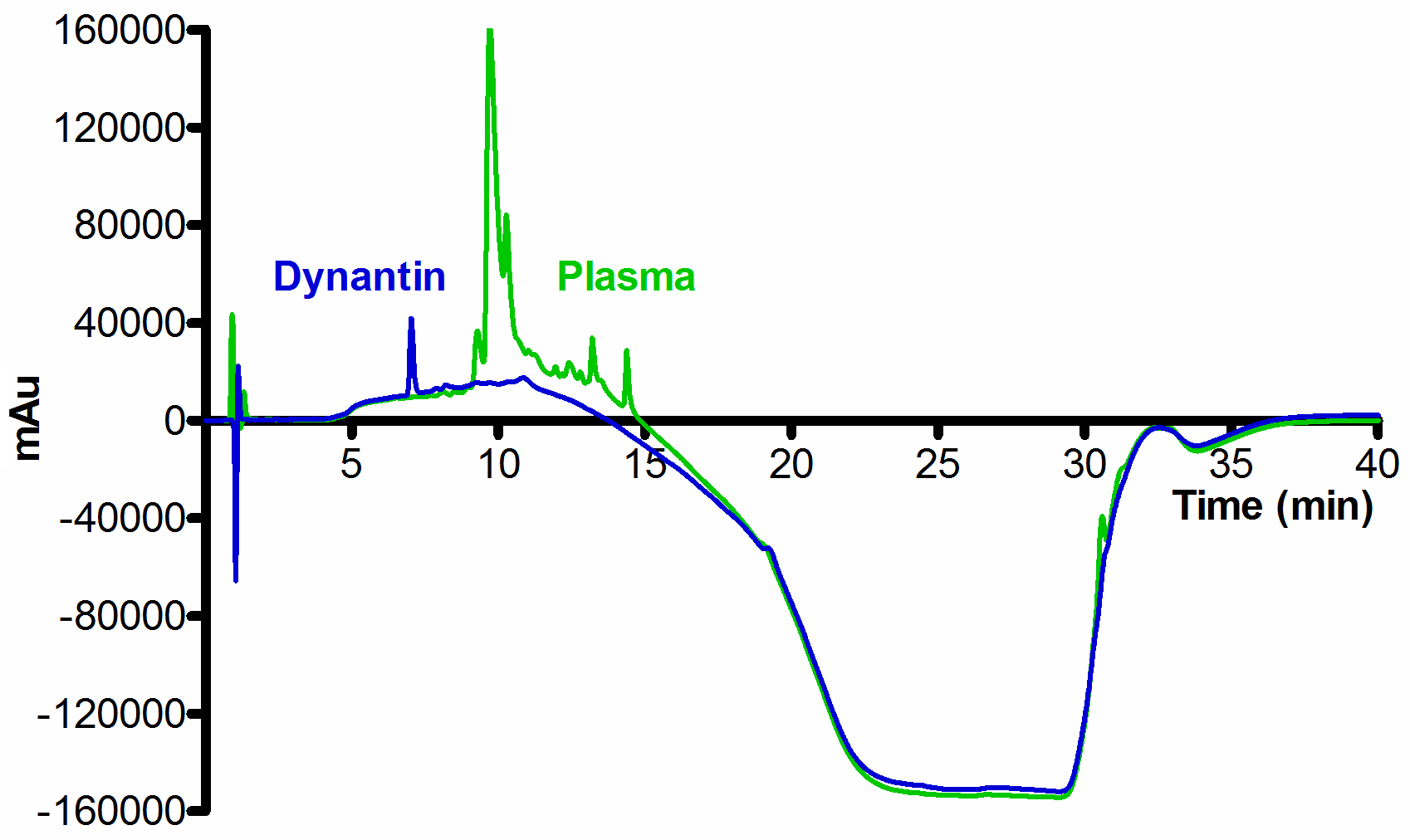

Supplement: S1 Fig — Dynantin (blue, 25 ng/μL in ddH2O) or rat plasma (green) were analyzed by RP-HPLC in the presence of 0.1% trifluoroacetic acid and detected by absorbance at 210 nm (8 μL injections). (TIF) [file pone.0204472.s001.tif]

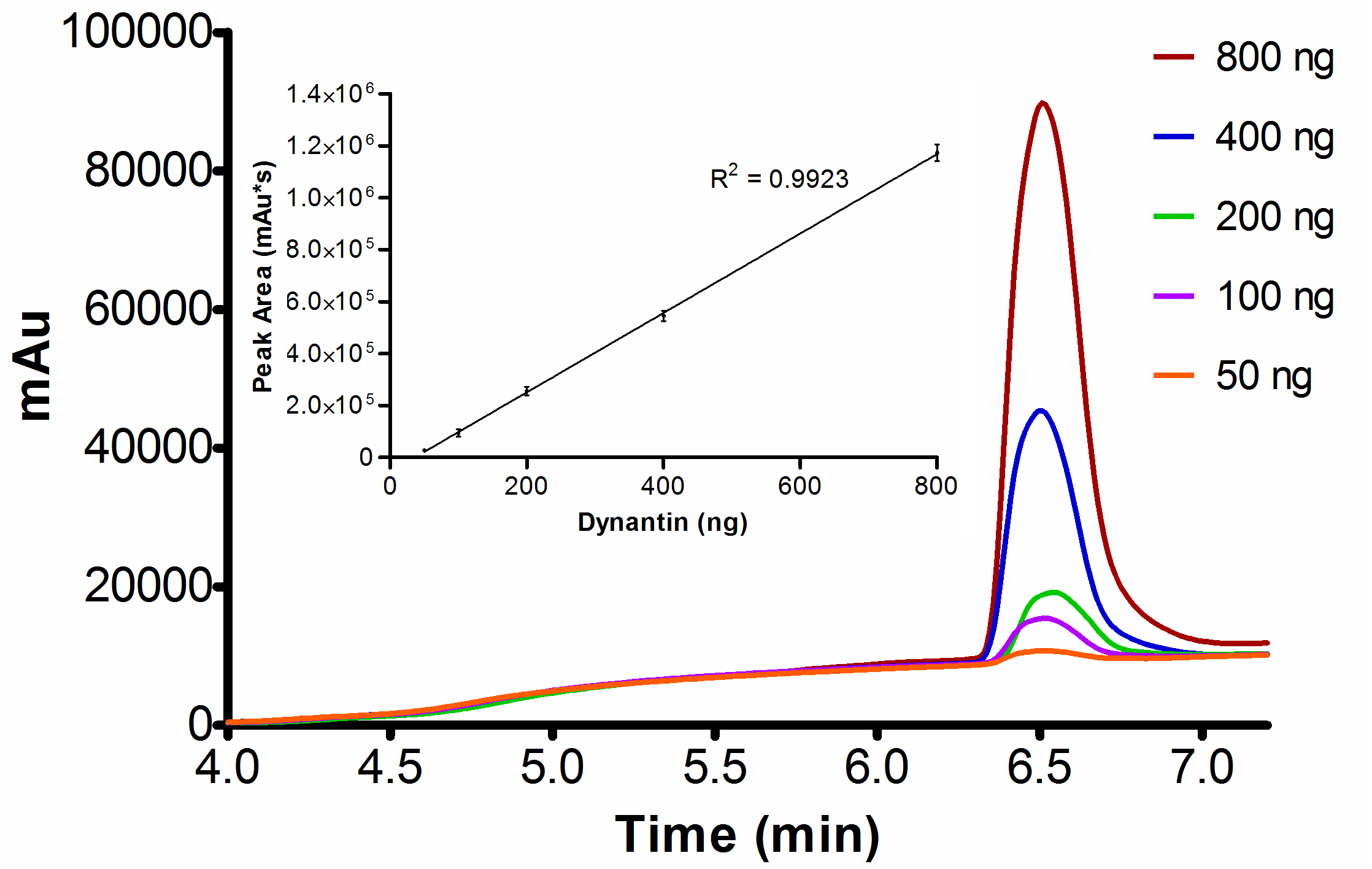

Supplement: S2 Fig — The Dynantin stock solution was diluted to various degrees in ddH2O and analyzed by RP-HPLC in the presence of 0.1% trifluoroacetic acid and detected by absorbance at 210 nm. Standard curve data shown is the average ± SEM of two separate experiments. (TIF) [file pone.0204472.s002.tif]

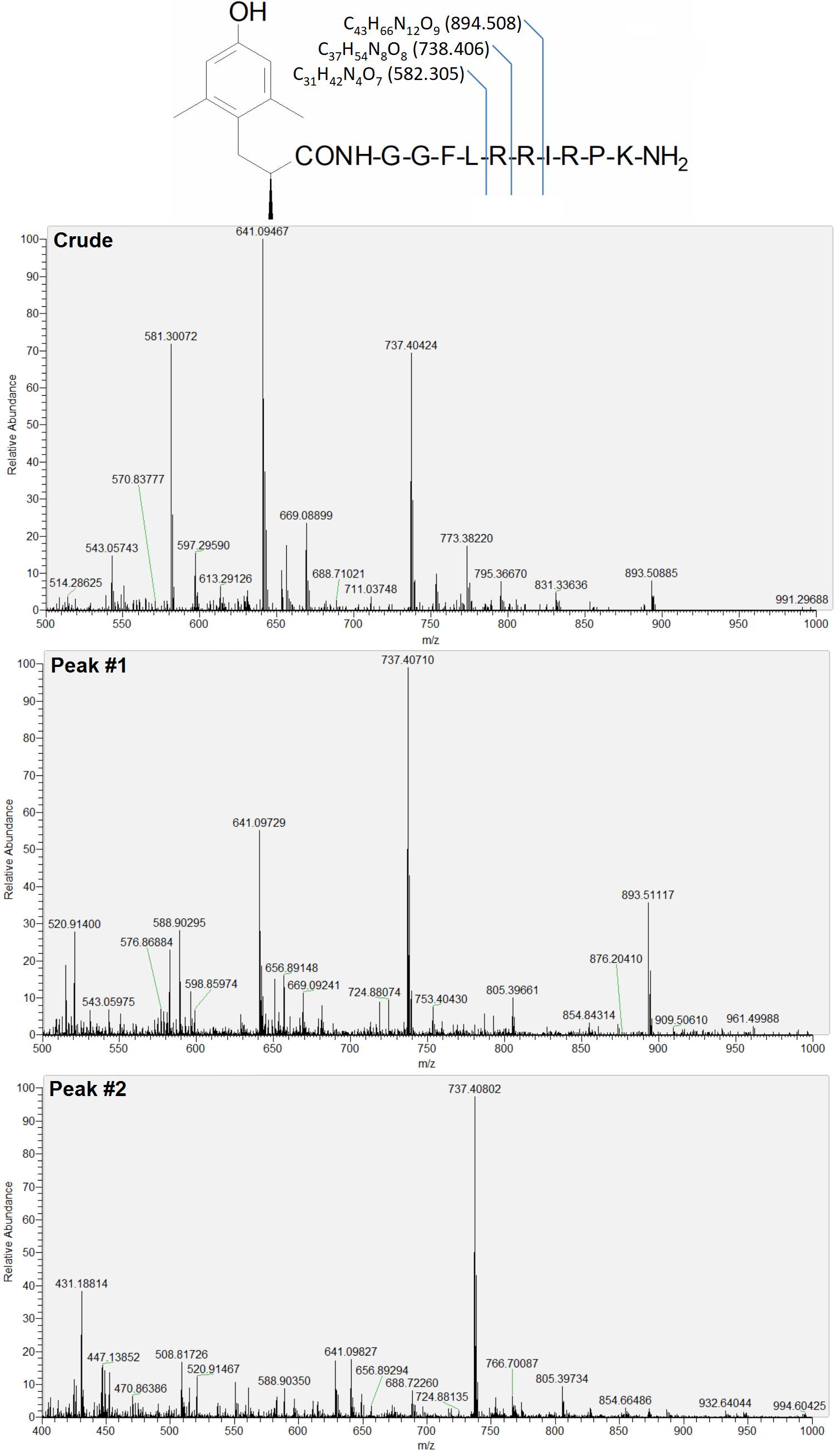

Supplement: S3 Fig — The plasma degradation products of dynantin were analyzed by direct injection electrospray ionization mass spectrometry in the negative ion mode as both the crude mixture (top) and individual fractionated peaks (middle and bottom), as outlined in Fig 2. (TIF) [file pone.0204472.s003.tif]

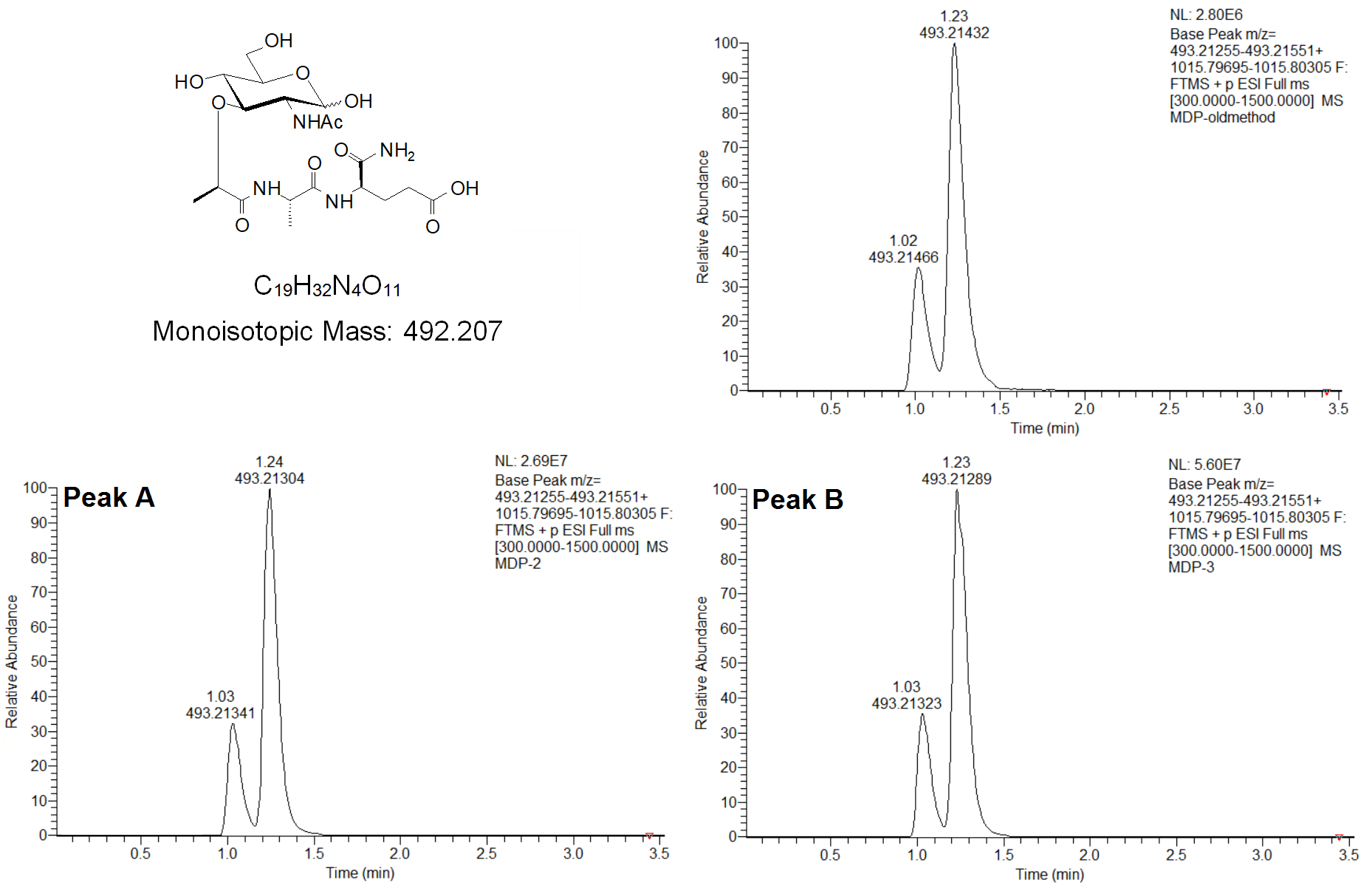

Supplement: S4 Fig — MDP was analyzed by positive mode electrospray ionization RP-HPLC-tandem mass spectrometry in the presence of 0.1% formic acid (top right). Individual MDP peaks, as outline in Fig 4 were also fractionated and subjected to the same analysis (bottom). (TIF) [file pone.0204472.s004.tif]
